# Strong Hereditary Predispositions to Colorectal Cancer

**DOI:** 10.3390/genes13122326

**Published:** 2022-12-10

**Authors:** Szymon Hryhorowicz, Marta Kaczmarek-Ryś, Emilia Lis-Tanaś, Jakub Porowski, Marcin Szuman, Natalia Grot, Alicja Kryszczyńska, Jacek Paszkowski, Tomasz Banasiewicz, Andrzej Pławski

**Affiliations:** 1 Institute of Human Genetics, Polish Academy of Sciences, Strzeszyńska 32, 60-479 Poznań, Poland; 2 Department of General and Endocrine Surgery and Gastroenterological Oncology, Poznań University of Medical Sciences, Przybyszewskiego 49, 60-355 Poznań, Poland

**Keywords:** CRC, CS, FAP, HNPCC, JPS, MTS, NAP, nonpolyposis, PJS, polyposis

## Abstract

Cancer is one of the most common causes of death worldwide. A strong predisposition to cancer is generally only observed in colorectal cancer (5% of cases) and breast cancer (2% of cases). Colorectal cancer is the most common cancer with a strong genetic predisposition, but it includes dozens of various syndromes. This group includes familial adenomatous polyposis, attenuated familial adenomatous polyposis, *MUTYH*-associated polyposis, *NTHL1*-associated polyposis, Peutz–Jeghers syndrome, juvenile polyposis syndrome, Cowden syndrome, Lynch syndrome, and Muir–Torre syndrome. The common symptom of all these diseases is a very high risk of colorectal cancer, but depending on the condition, their course is different in terms of age and range of cancer occurrence. The rate of cancer development is determined by its conditioning genes, too. Hereditary predispositions to cancer of the intestine are a group of symptoms of heterogeneous diseases, and their proper diagnosis is crucial for the appropriate management of patients and their successful treatment. Mutations of specific genes cause strong colorectal cancer predispositions. Identifying mutations of predisposing genes will support proper diagnosis and application of appropriate screening programs to avoid malignant neoplasm.

## 1. Introduction

Cancer, after cardiovascular diseases, is the second most common cause of death worldwide [1]. According to the World Health Organization (WHO) data, in 2020, cancer was diagnosed in 18.1 million patients (of which 9.3 million were men and 8.8 million were women), leading to 9.9 million deaths.

Colorectal cancer (CRC) is the third most common neoplasm and comprises 1.9 million cases (around 10.7% of all cancers) and 935,000 deaths in 2020. [2]. Colorectal cancer incidence rates in Europe show that males have a higher incidence, accounting for 35-42 cases, while females account for 24–32 cases per 1000 people. Moreover, the number of cases in developed countries is five times higher than in developing ones, even though developed countries boast lower mortality rates. In the case of developing countries, the ratio of mortality to detected cases remains high [3].

Around 70% of CRCs originate from spontaneous point mutations in oncogenes, tumor suppressor genes, and genes related to DNA repair mechanisms [4]. The remaining 30% are inherited mutations, from which 5–6% concern genes that show a strong predisposition to CRC occurrence [5]. Knudson’s hypothesis [6], first formulated in 1971 during an analysis of retinoblastoma, states that in the case of the inherited point mutation in one allele, the chances of a second, spontaneous mutation that leads to carcinogenesis are higher. This hypothesis has relevance to families whose members carry such mutations and thus may be strongly predisposed to developing CRC, 

Hereditary non-polypoid colorectal cancer (HNPCC) is a disease with a strong family history of CRC and neoplasms of this group, but the diagnosis is based on meeting the criteria (Amsterdam II) without identifying the genetic background. In 1999 criteria for determining families/persons with HNPCC were set by the National Cancer Institute (NCI to provide regular medical examinations and counselling [7,8]. The NCI also developed an alternative set of criteria (Bethesda, revised) which can be used to identify HNPCC individuals. However, their primary use is to determine whether discovered tumors should be tested for microsatellite instability (MSI) [9]. These criteria can apply to some non-HNPCC patients as well [10]. According to Mendel’s model, strong genetic predispositions to CRC are inherited with very high penetration, up to 100%. Among these nonpolyposis syndromes, the most common disease is Lynch syndrome (LS), and the rarest is Muir–Torre syndrome (MTS). The latter is less limited in symptoms and usually leads to the development of a wider variety of malignancies, such as sebaceous adenoma, sebaceous epithelioma, sebaceous carcinoma, or keratoacanthoma [11].

**Figure 1:**
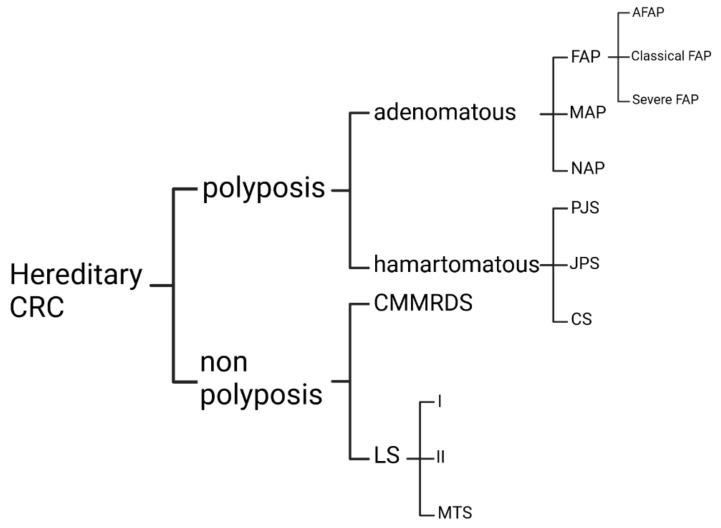
Classification of hereditary colorectal cancers (CRC) [13,14,15]. Abbreviations are as follows: FAP—familial adenomatous polyposis, LS—lynch Syndrome, MAP—*MUTYH* (MYH)-associated polyposis, MTS—Muir–Torre syndrome, NAP—*NTHL1*-associated polyposis, PJS—Peutz–Jeghers syndrome, JPS—juvenile polyposis syndrome, CMMRDS—constitutional mismatch repair deficiency syndrome, CS—Cowden syndrome, AFAP—attenuated familial adenomatous polyposis.

The CRC syndromes can be subdivided into nonpolyposis and polyposis entities, the most common of which are Lynch syndrome and familial adenomatous polyposis. Familial adenomatous polyposis (FAP) is characterized by numerous adenomatous polyps in the large intestine and, if left untreated, leads to malignant CRC development [12]. Besides FAP, the group comprises *MUTYH*-associated polyposis (MAP), *NTHL1*-associated polyposis (NAP), Peutz–Jeghers syndrome (PJS), juvenile polyposis syndrome (JPS), and Cowden syndrome (CS). They cause 1% of diagnosed CRC cases worldwide [13] (Figure 1).

Among the pathogenetic mechanisms leading to genomic instability, we can distinguish the previously mentioned MSI, chromosomal instability (CIN), and CpG island methylator phenotype (CIMP).

The CIN pathway, most widely described, accounts for about 80–85% of all CRC cases [16]. It is characterized by an imbalance in chromosome number, leading to aneuploid tumors and loss of heterozygosity (LOH). Mechanisms underlying CIN include changes in chromosome segregation, telomere dysfunction, and DNA damage, affecting critical genes that maintain correct cell function, such as adenomatous polyposis coli (*APC*), *KRAS, PI3K*, and *TP53*, among others. The *APC* mutations cause β-catenin translocation to the nucleus and enhance the transcription of genes that promote cell division. In contrast, mutations in *KRAS* and *PI3K* lead to sustained activation of MAP kinase, thereby increasing cell proliferation. Finally, loss-of-function mutations in the *TP53* gene, which encodes the p53 protein, result in a complete loss of cell cycle control and apoptosis capacity [17].

The MSI pathway is caused by a hypermutation phenotype resulting from a loss of DNA mismatched base repair (MMR). The ability to repair short DNA chains or tandem repeats (two to five base pairs) is reduced in tumors with microsatellite instability; therefore, mutations tend to accumulate in these regions. These mutations can affect both coding and non-coding regions involving, among other things, microsatellites. The initiation and onset of cancer originate in tumor suppressor genes and proto-oncogenes. Mutations in genes involved in MMR are a prevalent cause of MSI [18]. The MMR pathway is highly evolutionarily conserved and is responsible for both the correct pairing of single bases and the removal of insertion/deletion loops caused by polymerase slippage in highly repetitive regions. Polymerase slippage is the most common cause of such errors, but they also occur due to oxidative stress, base deamination, or methylation [19]. When no mutations are identified in genes linked to the MMR pathway, the MutSα complex, which consists of the *MSH2* and *MSH6* proteins, recognizes the mispairing or insertion/deletion. The complex is activated by ATP hydrolysis, which changes its conformation and recruits the MutLα complex consisting of *MLH1* and *PMS2* proteins. This tetrameric complex glides along the DNA, looking for unpaired sites on the newly synthesised strand, and when it finds them, it activates the RFC and PNCA proteins by triggering endonuclease activity and cutting the newly synthesised strand. Next, exonuclease 1 removes the synthesized DNA around the unpaired fragment. Finally, DNA polymerase delta synthesizes this strand fragment, and ligase 1 binds the pieces together [20].

Notably, the second function of the MMR pathway is to trigger a signaling cascade that leads to cell cycle arrest and apoptosis. Unfortunately, the model presenting this pathway’s work has not been fully elucidated [21]. It is apparent, however, that any errors in genes related to the MMR pathway are crucial in carcinogenesis. That also explains why mutations in the MMR pathway result in mutations in repetitive fragments and correlate strongly with the occurrence of MSI. For example, cancers characterized by MSI often exhibit mutations in the *MLH1*, *MSH2*, *MSH6*, *PMS1*, and *PMS2* genes [13].

The last of the three main pathogenic factors, CIMP, is caused by epigenetic instability. A common feature of CIMP tumors is the hypermethylation of oncogene promoters, leading to their silencing and subsequent loss of protein expression. Point mutations and abnormal methylation are two factors that interact in the development of CRC [22]. Genetics and epigenetics are not mutually exclusive in promoting the development of CRC in the presence of BRAF mutations and MSI in many CIMP tumors [23]

## 2. Materials and Methods

We searched the PubMed and Google Scholar databases for papers that examined hereditary CRC syndromes. Most of the reports included in the analysis have been published during the last 30 years. The keywords colorectal, adenoma, cancer risk, familial adenomatous polyposis, *MUTYH*-associated polyposis, Muir–Torre syndrome, *NTHL1*-associated polyposis, Peutz–Jeghers syndrome, juvenile polyposis syndrome, Cowden syndrome, and attenuated familial adenomatous polyposis were used, among others.

## 3. Results

### 3.1. Nonpolyposis CRC Predisposition

Here, HNPCC is not synonymous with LS because identifying germline mutations in DNA mismatch repair (MMR) genes led us to distinguish LS from other conditions associated with familial colorectal cancer. The diagnosis of HNPCC is based on the Amsterdam II criteria, which are as follows: having at least three relatives who have had one of the LS-related cancers in their lifetime, and (1) one of them should be a first-degree relative of the other two, (2) two consecutive generations developed cancer, (3) at least one person developed cancer before the age of 50, (4) familial adenomatous polyposis has been ruled out, and (5) tumors have been verified to be cancerous [8].

In the HNPCC group of cancer, we can observe cancers demonstrating defective DNA MMR with MSI and cancers demonstrating intact DNA MMR. Generally, HNPCC is defined by family history, whilst LS and constitutional mismatch repair deficiency syndrome (CMMRDS) are characterized by mutations in specific, known genes (mentioned later in the text). In patients with HNPCC with excluded mutations in MMR genes, the risk of extra CRC is significantly lower than in cases of LS [24].

**Figure 2:**
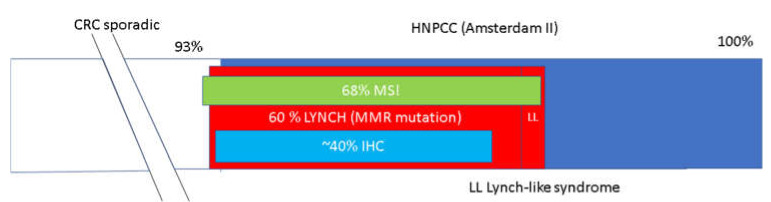
Nonpolyposis colorectal cancer (CRC) [25,26,27]. Abbreviations are as follows: HNPCC—hereditary nonpolyposis colorectal cancer; IHC—immunohistochemistry; LLS—Lynch-like syndrome; LS—Lynch syndrome; MMR—mismatch repair; MSI—microsatellite instability.

In the study of patients with HNPCC syndrome, it was observed that MSI is present in 70% of cases, and the abnormal expression of MMR genes, which is diagnosed by immunohistochemistry (IHC), tests in about 40% of HNPCC cases. Among the cases with MSI, the majority of them are LS cases with a mutation in DNA repair (MMR) genes detected, although MSI may also be observed in sporadic CRC due to somatic alteration in MMR genes or cases of Lynch-like syndrome caused by mutation of other genes (*POLE/POLD1*). A subset of LS cases does not meet the Amsterdam II criteria despite detected mutations in MMR genes (Figure 2). That is because less than 2–3% of MMR genes mutations occur de novo, and no family history is observed. The penetration of MMR gene mutations does not reach 100%. Additionally, a small number of offspring or lack of progeny, especially in highly developed countries, may contribute to the lack of the fulfilled Amsterdam II criteria [25,26,27].

#### 3.1.1. Lynch Syndrome

Autosomal dominantly inherited LS is a strong predisposition to malignancies, most commonly CRC and endometrial cancer. The estimated lifetime risk is 50–70% and 40–60%, respectively. In addition, the average age of a patient with Lynch syndrome who develops intestinal cancer is 45 years, with an 80% chance of developing it during their lifetime. Polyps occur sporadically. The period for cancer to form from an adenoma is one to three years, much faster than spontaneous cancer, which takes 8 to 17 years for the same transformation to occur. The CRCs originating from LS are characterized by rapid synchronicity acquisition and quick metastasis. It is estimated that after developing CRC, the chance of having another cancer is 30% after 10 years and 50% after 15. Furthermore, LS can additionally lead to the development of other cancers—the stomach (7%), urinary tract (3%), ovary (9%), biliary tract (3%), small intestine, brain (3%), pancreas (4%), and skin [28]. A syndrome closely related to LS is the MTS, which is also characterized by skin lesions, particularly sebaceous adenomas and carcinomas, epitheliomas, and squamous cell keratoses [29,30].

The diagnosis of Lynch syndrome is based on the identification of mutations in MMR genes (*MSH2*, *MLH1*, *MSH6*, and *PMS2*). Nowadays, the development of sequencing techniques allows the sequencing of many genes at a rapid pace and is relatively cheap. Indeed, MSI assay (MSA) or immunohistochemistry (IHC) analysis can determine the contribution of MMR mutations or even indicate the loss of specific gene activity, which allows further optimization of mutation search studies [31].

The development of CRC itself in LS patients begins with normal colonic epithelium, in which all cells (arising from the germline) encode heterozygous mutation in one of the genes associated with the MMR pathway. At this point, polyps smaller than 8 mm can form, whose MMR pathways and microsatellites are still stable [32]. Their formation is most likely related to a mechanism mediated by APC [33]. Furthermore, probably by haploinsufficient genes linked to the MMR pathway or somatic mutations, biallelic loss of function in MMR genes occurs, resulting in DNA error propagation and MSI. This phenomenon occurs in all polyps larger than 8 mm and some smaller than 8 mm [32]. Such pathologies accumulate and lead to somatic mutations, including reading frame alterations in genes, such as *APC* [34], *TGFBR2* [35] or *BAX* [36], which results in an accelerated process of polyp-to-cancer transformation in large polyps. The CRC formed from this polyp is characterized by the loss of MMR and MSI presence [33].

Furthermore, the proteins formed due to the reading frame alteration mutation attract lymphocytes involved in the response against cancer (tumor-infiltrating lymphocytes). On the other hand, the hypothesis assuming that polyps larger than 8 mm are mainly responsible for the tumorigenesis process can not explain the findings of Ahadova A. and his team [37], which detected intestinal crypts located near adenomas without MMR activity that lack MMR-associated protein expression but have not yet undergone tumorigenesis. According to this hypothesis, precursors are not crucial in forming polyps [37]. Additionally, collected data suggest that these widely presented cells, with an inactive MMR pathway, are characteristic of the epithelium in LS patients [38] and can acquire somatic mutations in *TP53* or *CTNNB1* and, thus, rapidly initiate tumorigenesis [37,39,40].

The first mention of LS came in 1913, when Warthin A.S. published a family tree of the so-called “G family”, clearly indicating that there was a hereditary causative factor for cancer [41]. The next breakthrough that led to a better understanding of this type of cancer was the work of Lynch H.T. and his team [42]. He succeeded in ruling out that the cancer was related to FAP. The syndrome was named cancer family syndrome (CFS), and despite the scientific community’s disapproval of the genetic etiology of CFS, research began. Subsequent years brought additional observations, such as the isolation of a subtype of CFS, termed MTS [43], and the subsequent creation of terminology distinguishing LS and MTS.

The first genetic locus responsible for LS was found on chromosome 2p21 using polymorphic microsatellite repeat markers [44]. A second genetic locus responsible for LS has been found on chromosome 3p21-23 in members of families with MSI-associated cancers [45]. However, not all LS families showed linkage to these loci, indicating more significant genetic heterogeneity in the etiology of LS. Genes specifically responsible for LS as *MLH1, MSH2, MSH6,* and *PMS2*, and located near *MSH2,* the *EPCAM* gene, were found thanks to modern screening methods [18]. According to the InSiGHT database, the frequency of LS-associated mutations is 42% for *MLH1*, 33% for *MSH2*, 18% for *MSH6*, and 7.5% for *PMS2* [46]. Early studies of the *MLH1* and *MSH2* genes revealed that most LS patients (60%) have large deletions in exon regions in these genes [47]. Moreover, a high frequency of rearrangements within the *MSH2* gene, particularly mainly deletions and duplications, has been shown to lead to loss of protein function [48].

It is worth mentioning that the course of LS is quite variable since mutation in the germline can affect as many as five different genes. As mentioned earlier, the most common is the “classic” variant caused by mutations in *MLH1* and *MSH2* [7], in which the disease surfaces around 43–46 years of age with tumors characterized by MSI. Interestingly, 50% of tumors developing in LS patients with *MLH1* mutation also have a somatic mutation in *CTNNB1*. Compared to that, *MSH2* variants of LS usually (75% of *MSH2* cases) develop tumors with mutations in the *APC* gene [49]. It is worth mentioning here that mutations in *MSH2* cause an increased chance of developing tumors outside the colon, including MTS, one of the variants of LS [50]. 

On the other hand, the atypical form of LS is strongly associated with mutations in *MSH6* and *PMS2* [51]. Patients with the *MSH6* variant of LS all exhibit somatic *APC* mutations in tumors, and none exhibit somatic *CTNNB1* mutations, according to a study on a Finnish population [49]. Individuals with a mutation in *MSH6* have an increased chance of endometrial cancer with an age of onset higher than 50 years [51]. These tumors do not necessarily manifest MSI [52], but they harbor mutations due to unpaired DNA bases [53]. In the case of *PMS2* gene mutations, carriers most often develop CRC, but later than usual [54]. The differences in the development of the “typical” LS compared to its second variant might be since both *MSH6* and *PMS2* are partially functional duplicates of *MSH3* and *MLH3*, where neither *MLH1* nor *MSH2* is crucial for the proper functioning of the entire pathway.

When it comes to risk reduction, for the general population, the colonoscopy with polypectomy every 10 years significantly reduces the risk and improves survival via early detection, but in LS patients, the situation is more complex. A study by Engel et al. [55] shows that many people affected by LS develop CRCs despite colonoscopic surveillance. On the one hand, Jarvinen et al. [56] showed that CRC risk halves if a colonoscopy is carried out every 3 years in cases of LS. On the other hand, LS patients with regular colonoscopies still have a 15% risk of developing CRC in 10 years [57]. In fact, CRC was the most frequently observed cancer in those patients [58]. The CRC risk and colonoscopy efficiency depend on the affected genes. To be precise, patients with LS associated with either *MLH1* or *MSH2* have a lifetime risk of CRC of around 50% despite colonoscopic surveillance.

Meanwhile, patients with LS associated with *MSH6* and *PMS2* mutations have lower CRC risk in their lifetime, which surveillance can further reduce [59]. On the other hand, while patients with LS associated with *MSH6* have a lower CRC risk, the risk of developing adenomas is more significant in this group, aside from *MSH2* patients, where the risk of advanced adenomas is the highest. The LS patients associated with *MLH1* do not develop adenomas as frequently despite having a high CRC risk. That suggests that *MLH1*- and *MSH2*-associated CRC development happens under different pathways. *MLH1* is primarily associated with somatic *CTNNB1* mutations, while *MSH2* is associated with somatic *APC* mutations, which might explain why *MSH2*-associated cancers exhibit quick transformation from adenomas to carcinomas with MMR deficiency, while *MLH1*-associated cancers usually progress without polyp formation. As for *MSH6*, while it has a higher proportion of somatic *APC* mutations compared to CTNNB1 mutations, its incidence rate (compared to both *MLH1*- and *MSH2*- associated CRCs) is low [49]. Engel et al. hypothesised that *MSH6* causes incomplete MMR deficiency, primarily associated with mononucleotide repeats, which, in turn, lowers the likelihood of driver mutations. This hypothesis might explain the high success rates of screening *PMS2* carriers. Adenomas in patients with *PMS2*-associated CRCs do not exhibit *CTNNB1* mutations; consequently, those carriers have a lower CRC risk.

Explanations as to why colonoscopy with polypectomy is not as satisfactory in the prevention of the development of CRCs in people with LS were pursued by Ahadova’s [57] team. Five hypotheses were made to explain why CRC can still develop despite triennial colonoscopies. The simplest hypothesis is that colonoscopic surveillance fails to identify and remove adenomas. There is precedent for this because one of the newest meta-studies has shown that the adenoma miss rate is as high as 33% in patients with increased CRC risk [60]. Despite that, no studies could show whether optimization of colonoscopy could reduce the occurrence of CRC in patients with LS [57].

The second hypothesis postulates that a possible reason for occurrences of CRCs stems from accelerated progression from adenoma to carcinoma. Compared to 10 or more years in the general population, the CRC progression from benign polypoid precursor to cancer is accelerated [61]. That seems contradictory with other findings because while triennial colonoscopic surveillance halved the risk of developing CRC, increasing the examination frequency to annual colonoscopies did not improve detection rates [62], which is especially true for CRCs associated with mutations in either *MLH1* or *MSH2*. This evidence points to the fact that CRCs associated with either of these genes undergo a different pathway leading to tumorigenesis. That is, in fact, a third hypothesis for the challenges of colonoscopic surveillance. There is evidence for undetectable precursor lesions and routes to cancer without the adenoma stage [57]. These lesions are impossible to detect using routine colonoscopy and are only detectable via MMR protein staining [63]. Moreover, current data points to the fact that the sequential model of CRC progression from adenoma as we know it is oversimplified. Because of that, Ahadova et al. urge us to consider at least three possible pathways, as follows: (a) progression from an adenoma with secondary inactivation of the MMR system, (b) progression from an initially MMR-deficient adenoma, and (c) progression from MMR-DCF directly to invasive cancer without adenoma formation [57].

The following two hypotheses were created to explain why more frequent colonoscopies result in a higher number of lesions than less regular examinations. The first hypothesis, based on data, points out that not all lesions develop into CRCs. Because of that, more frequent surveillance discovers pre-cancerous developments routinely cut out, although they could regress over time and eventually disappear entirely [64]. Mentioned data points out that LS-associated cancers are highly immunogenic, which results in the generation of FSPs that can elicit strong immune responses and cause in vitro killing of FSP-expressing cells by T cells [65]. The second hypothesis suggests that this is a colonoscopy which might play a role in the pathogenesis of CRC. It is controversial, but two potentially cancerogenic, colonoscopy-associated factors can be listed. Firstly, colonoscopy preparations affect the microbiome of the bowel [66]. Secondly, the process can irritate bowel epithelium—the endoscope and pressure it enforces onto the bowel can create micro-injuries that damage the mucosa if biopsies are performed. These micro-injuries could, in turn, initiate/accelerate tumorigenesis. There exists a study supporting these claims [67]. The risk listed is low (0.3–0.6%), but data suggest that highly frequent colonoscopies can indeed contribute to lesion count.

Strides are being made in the prevention/treatment of LS-associated cancerogenesis. A study by Burns et al. [68] shows that orally taking 600 mg/day of cyclooxygenase-2 inhibitors (in this case aspirin) for over 2 years reduces incidence rates of CRC associated with LS, but additional studies are needed to explain this interaction. As for treatment, the fact that LS-based CRCs are highly immunogenic can be exploited. The team of Le [69] targeted programmed death receptor 1 (PD-1) with monoclonal antibodies (pembrolizumab) as a way to manipulate the patient’s immune system and proved to increase disease control. Treatment of metastatic MMR-deficient CRCs showed better outcomes with a lower hazard ratio of progression than those of MMR-proficient CRCs. This study indicated a potential treatment option, although the authors did not focus on LS specifically.

#### 3.1.2. Constitutional Mismatch Repair Deficiency Syndrome

It is known that CMMRDS is a recessive, rare cancer predisposition caused by biallelic mutations in MMR genes [15]. Depending on the MMR gene in which the mutation occurred, four types of this disease can be distinguished [70]. The most frequently presented in CMMRDS are hematologic malignancies, brain/central nervous system tumors, and LS-associated tumors, such as colorectal cancers. These neoplasms develop mainly in children and young adults before 18. The mean age of diagnosis is 6, 9, and 17 years, respectively [15]. It is estimated that CMMRDS occurs once every 1,000,000 live births [71]. Generally, the CMMRDS phenotype overlaps with the neurofibromatosis type 1 (NF1) phenotype, mainly manifested by the presence of multiple hyperpigmented skin areas called café-au-lait macules (CALMs). Most CMMRDS patients share this trait but not all. However, there are no reports of germline mutations of the NF1 gene in patients, no matter the phenotype [15,72].

As mentioned earlier, there are four types of CMMRDS, depending on which gene harbors the germline mutation.: CMMRDS1 for the *MLH1* gene, CMMRDS2 for the *MSH2* gene, CMMRDS3 for the *MSH6* gene, and CMMRDS4 for the *PMS2* gene [70]. These are the same genes already mentioned in this review’s LS section. Contrary to LS, both gene copies must be mutated for the disease manifestation. When only one allele is being mutated, the person is considered a carrier due to the recessive nature of this syndrome [15]. In other words, patients suffering from LS are simultaneously regarded carriers for the CMMRDS. Because of this, the name “Lynch Syndrome III” was proposed but was ultimately disregarded [73]. The first two types of CMMRDS share more similarities than the remaining two and, thus, can be considered a single group, depending on the circumstances. The most common malignancies are those of the central nervous system (53.5% of patients)—they are most frequent among *PMS2* patients (60%), then *MSH6* (55%) and *MLH1*/*MSH2* (34%). Next are LS-associated malignancies (40% of patients), with *PMS2* (46%), *MLH1*/*MSH2* (37%), and *MSH6* (28%). Finally, the least common malignancies out of the three listed are hematological ones (31% of patients), with *MLH1*/*MSH2* (44%), *MSH6* (34%), and *PMS* (25%) [15]. A highly defective MMR system leads to a high frequency of mutations in somatic cells, including mutations in housekeeping and cell cycle control genes leading to cancerogenesis.

For the suspected diagnosis of CMMRDS, a scoring system was developed. The criteria list multiple malignancies, premalignancies and other features. Each of them is assigned a score ranging from 1 to 3 points. The patient is given a score based on these factors (or lack thereof). A score of 3 or higher indicates the need for CMMRDS testing. Some tumors are particular and characteristic of this disease entity, so they were assigned 3 points. Following these criteria’s rules, such patients need thorough testing regardless of the presence of additional non-neoplastic features characteristic of CMMRDS [15].

The CMMRDS treatment is based on checkpoint inhibitors, which belong to the group of immunomodulators. These compounds inhibit protein activity, impeding the immune response to cancer [71]. For example, it has been demonstrated that the blockage of the interaction between PD-1 and PD-L1 proteins has an effective clinical effect in patients with glioblastoma multiforme [74]. This approach is used in a nivolumab antibody to treat, among others, metastatic melanoma [75]. Furthermore, vaccination with tumor antigens (neoantigens) may be another promising strategy in CMMRD patients [71].

### 3.2. Adenomatous Polyps

#### 3.2.1. Familial Adenomatous Polyposis

Familial Adenomatous Polyposis is a disease inherited in an autosomal dominant manner that is characterized by the presence of hundreds to thousands of adenomatous polyps localized mainly in the mucosa of the colon and rectum [76]. These polyps can coexist with fundic gland polyps (FGPs) and polyps in the duodenum [77]. In the classic form of the disease, the first polypoid lesions usually appear in the second decade of life, which means that in half of FAP patients, the first polyps are present as early as in the 15^th^ year of life. By age 35, almost 95% of patients present them [78]. Nevertheless, cases of FAP have been reported in a 5-year-old child [79] and even a 3-year-old child in the Polish population [80]. FAP occurs de novo in 1 per 8000–10,000 live births [81] and accounts for approximately 1% of all colorectal cancers [82], placing it the second most common CRC syndrome = after LS [83]. In FAP patients, the risk of developing colorectal cancer by age 40, in the absence of timely diagnosis and treatment, is almost 100% [81]. In addition to CRC, patients with FAP also have an increased risk of developing other cancers, such as duodenal cancer [84], thyroid cancer [85], hepatoblastoma [86], pancreatic cancer [87], brain cancer [88], or adrenal adenoma [89]. Other common extracolonic manifestations include osteomas, skin tumors, soft tissue tumors (desmoids) [90], jawbone abnormalities [91], or congenital hypertrophy of the retinal pigment epithelium (CHRPE) [92]. Of these, CHRPE occurs most frequently (up to about 90% of FAP patients) [93], while desmoids are the most common cause of death, with a frequency of 10-20% [94,95].

A family cancer history is an essential aspect of diagnosis. If there is a family history of CRC or the patient reports symptoms, such as rectal bleeding or abdominal pain, a sigmoidoscopy or colonoscopy should be performed. The primary diagnosis is based on clinical evaluation and endoscopy or complete colonoscopy. A complementary approach is genetic testing used for early detection and confirmation of causative factors of FAP. *APC* and *MUTYH* are conferred as the main predisposing genes for FAP and recommended for molecular diagnosis. [96]. Genetic counseling should be offered to first-degree relatives of FAP patients, especially between the ages of 10 and 12. Patients with a mutation in the *APC* gene but no apparent signs of disease should undergo sigmoidoscopy or colonoscopy annually [97,98].

The classic form of FAP results from germline mutations in the *APC* gene located on the long arm of chromosome 5 in the q21–q22 region [82,99]. The *APC* is a suppressor gene that encodes a protein involved in the Wnt signaling pathway. APC protein regulates the level of β-catenin, which activates the expression of genes related to cell division, such as *c-myc*. Loss of APC function leads to a loss of control over cell proliferation [100,101].

The *APC* gene mutations occur in about 60–85% of FAP patients. In most cases, they are small insertions or deletions, the most common of which include the AAAGA deletion at codon 1309 and the ACAA deletion at codon 1061, referred to as mutational hot spots [102,103,104]. Mutations in the *APC* gene are usually inherited. However, about 25% of FAP patients develop them de novo [55]. In these patients, the diagnosis is generally made about 10 years later and occurs when CRC symptoms have already developed [105].

#### 3.2.2. FAP Classification

Disease severity and the presence of extracolonic manifestations are correlated with the location of the mutation in the *APC* gene. Based on these features, FAP can be classified into three phenotypes: mild, intermediate, and severe, with severe and intermediate phenotypes constituting the classic form of FAP. 

The severe phenotype is characterized by the presence of more than 1000 polyps. The disease manifests at a young age—mainly between the first and second decades of life—while the average age of developing colorectal cancer is about 34 years. APC protein truncating mutation between codons 1250 and 1464 of the *APC* gene has been detected in this phenotype. In this case, multiple extracolonic manifestations are observed [106,107].

For the intermediate phenotype, the range of the number of polyps is not clearly defined but is assumed to be hundreds to thousands of polyps developing in the second and third decades of life [107,108]. The average age of developing colorectal cancer in untreated individuals is about 40 years [76]. Most of the germline mutations in the *APC* gene causing the intermediate phenotype are located between codon 157 of exon 4 and codon 1595 of exon 15, excluding the mutation cluster region (MCR) [107,109,110,111,112,113].

Attenuated FAP is a less aggressive variant of FAP. AFAP is characterized by the presence of fewer polyps (<100), their usually right-sided distribution excluding the rectum, later age of developing CRC (by 15 years), and a lower risk of developing CRC (not higher than 70%) compared to FAP [114,115]. Mutations in the *APC* gene associated with AFAP occur upstream of codon 157, downstream codon 1595, and in the alternatively spliced region of exon 9, and are thought to affect 10% of patients diagnosed with FAP. AFAP tends to have a reduced incidence of extracolonic manifestations but is often accompanied by other gastric and duodenal adenomas [116].

Although less common and less likely to dramatically increase FAP risk than *APC* mutations, inherited mutations in many other genes can also lead to polyposis and CRC. Such genes include *MUTYH* with the autosomal recessive inherited disease MAP (*MUTYH*-associated polyposis). The *MUTYH* gene encodes a DNA glycosylase involved in repairing oxidative DNA damage in the base excision repair (BER) system, thereby preventing G:C to A:T transversion in the *APC* gene [117]. The most common mutations in the disease entity include Y179C (previously referred to as Y165C) and G396D (previously referred to as G382D) [118]. In MAP, the number of polyps usually does not exceed 100, but unlike AFAP, they are often hyperplastic or sessile serrated. The average age of diagnosis is about 47 years [119,120], while the risk of developing CRC is 70% by age 70 [121].

Similarly to MAP, recessively inherited mutations in *NTHL1* have been linked to newly described adenomatous polyposis – NAP (*NTHL1*-associated polyposis). The *NTHL1* gene is involved in the BER system. Carriers of biallelic nonsense mutations in the *NTHL1* gene can develop both intestinal symptoms, but also multi tumor phenotype. Here, CRC occurs in about half of mutation carriers at an average age of 55. Duodenal polyps are observed sporadically. Among extraintestinal cancers, breast cancer occurs in about 55% of patients, while gynecological cancers occur in about 27% of patients. Urothelial and basal cell carcinomas are also frequently observed among carriers [122,123].

As mentioned earlier, the risk of developing CRC for FAP patients is almost 100% [81]. Therefore, surgical treatment is the only way to avoid cancer. There are two main procedures—total abdominal colectomy with ileorectal anastomosis (IRA) or total proctocolectomy with ileal pouch-anal anastomosis (IPAA). The first approach is recommended for AFAP patients and involves resection of the colon. The second approach is preferred for FAP patients and involves resectioning the large intestine with the rectum and forming a reservoir (“pouch”) from the ileum and its anastomosis with the rectum. We can also distinguish a total proctocolectomy accompanied by end ileostomy, leading to a permanent stoma. This operation is performed when IPAA is not advisable due to the tumour’s location and technical difficulties [124,125].

Significant variation in both age and extent of symptoms is seen among patients, and this variation is observed not only in carriers of the same mutation but even in members of the same family. That should be considered when planning treatments, and further research in the search for disease course modifiers should continue [126,127].

### 3.3. Hamartomatous Polyposis

Hamartomatous polyps (HP) are rare polyps that consist of regular, “healthy” tissues and mature, defined cells that are no different from those found in non-polyp structures. The critical feature of HPs is their abnormal cell number and/or location.

HPs occur sporadically in the general population, and the presence of such polyps in the gastrointestinal (GI) tract does not imply an increased risk of cancer development. However, numerous disease syndromes are characterized by the high presence of HPs in the GI tract and are associated with an increased lifetime risk of cancer development, including CRC, specifically hamartomatous polyposis syndromes. The three most common have been described below, namely Peutz–Jeghers Syndrome (PJS), Juvenile Polyposis Syndrome (JPS), and Cowden Syndrome (CS).

#### 3.3.1. Peutz–Jeghers Syndrome

Peutz–Jeghers Syndrome (PJS) is a hereditary disease characterized by mucocutaneous pigmentation (melanocytic spots) of fingers, lips, and mucosa of the nose, cheeks, and the formation of hamartomatous polyps (HPs) in the GI tract [128,129,130]. These polyps are usually multiform, and their surface is covered with papillae. In addition, there are branching strands of smooth muscle covered by a mucous membrane [131]. The first polyps appear during early teens (median age 11–13). Common symptoms include anemia, abdominal pain, rectal bleeding, and intussusception [132], which occur in half of the patients at various stages of life [133]. Patients with PJS have a significantly increased risk of developing cancers of the GI tract and other organs, including the pancreas (36%), breast (54%), lungs (15%), testes (9%), ovaries (21%), and uterus (9%) [134]. The disease prevalence is about 1 in 100,000 people, with estimates ranging from 1 in 8300 to 1 in 280,000, depending on the study [135].

The disease is inherited in an autosomal dominant manner. The vast majority of cases have been linked to a germline mutation at locus 19p13.3 in the serine/threonine kinase 11 gene (*LKB1*, also known as *STK11*) [136,137,138,139] consisting of 10 exons, 9 of which encode the protein. [137]. Minor mutations define most cases, but the involvement of larger-scale *LKB1* mutations, specifically DNA copy number variations (CNV), has also been discovered, with CNVs estimated to be responsible for about 30% of PJS cases [140,141]. In 2015, it was shown that deletion of exons 2–3 of the *LKB1* gene causing PJS always involved Alu elements—the most common transposons in the human genome, occurring at more than one million copies [142,143]. However, the gene mutation is not the exclusive scenario causing PJS; the presence and/or functionality of the LKB1 protein depend on a larger number of genetic and epigenetic factors, often still unexplored [141]. Inactivation of *LKB1* has been shown to affect approximately 91% of affected families [144]. It has been proven that LKB1 binds to the p53 protein and regulates specific apoptotic pathways, which categorizes it as a tumor suppressor protein [145]. Most cases of PJS are characterized by mutations resulting in a lack of *LKB1* expression (or, rarely, a lack of function), which leads to the deregulation of the cell cycle and allows cells to avoid apoptosis. Avoiding apoptosis is a gateway to developing pathologies (polyps) and carcinogenesis [146].

Diagnosis and treatment of PJS are mainly based on the early detection of small intestinal polyps and their removal to prevent blockage of the GI tract and/or cancer development. In the past, the most recommended method was intra-operative enteroscopy (IOE). This procedure combines laparoscopy and endoscopy and allows the surgeon to view the inside of the intestine through a small light source and a camera [147]. The HPs localized this way are surgically removed. Such a procedure allows for the removal of all polyps, but involves risks. The patient’s recovery is long, and complications can occur, mainly small intestine adhesions, which are abnormal formations that fuse loops of intestines together that were not originally connected. They can restrict the patency of the gastrointestinal tract and make potential, similar surgeries in the future more difficult [148]. In 2001 Yamamoto H. and his team [149] invented—double-balloon enteroscopy (DBE). It uses an enteroscope equipped with two latex balloons that can be controllably inflated with air or deflated. These balloons can also be moved (relative to the enteroscope), allowing the device and its camera to gradually move deeper into the intestine using carefully planned and controlled maneuvers [149]. Such a procedure is less invasive than IOE and allows for a more thorough examination of the intestine inside. Unfortunately, the presence of intestinal adhesions disqualifies the patient from the procedure, and IOE surgery is recommended instead.

In addition to treating PJS, research teams are striving to develop ways to prevent the disease or at least slow the onset of pathology. Studies using a mouse model of PJS have identified rapamycin as a potential chemopreventive agent, effectively reducing polyposis. It is an antibiotic with immunosuppressive and antiproliferative properties extracted from the fungus *Streptomyces hydroscopicus*. Oral application of the compound significantly reduced the number of HPs, their size, and their microvessel network density, which is suspected to be due to the anti-angiogenic effect of rapamycin [150,151].

#### 3.3.2. Juvenile Polyposis Syndrome

Here, JPS is a rare genetic disorder manifested by juvenile polyps (JPs) in the large intestine (from five to several hundreds of polyps) and other sections of the GI tract from the stomach to the rectum and is associated with a significantly increased risk of colorectal cancer and, to a lesser extent, other GI cancers [152]. Sporadic JPs are reported in 2% of the pediatric population and are unrelated to an increased risk of cancer development [95]. The cancer risk for patients suffering from JPS is challenging to assess unequivocally. In 1998, the risk of developing gastrointestinal cancer was estimated at more than 50% [153]. Then, in 2007, the lifetime risk of developing CRC was estimated at 38.7% [154]. The average age of CRC diagnosis is 42 years [153,154]. To diagnose JPS in a patient, they must meet at least one of the following criteria: ≥6 JPs in the colon and/or JPs occurring in different segments of the gastrointestinal tract and/or a family history of JPS and ≥1 polyp in the gastrointestinal tract [155]. Furthermore, JPS is thought to occur once every 16,000–100,000 live births [156].

The JPs are polyps ranging from 5 mm to 50 mm in diameter, characterized by a spherical, pedunculated shape with a lobular structure with traces of erosion. Swollen connective tissue (lamina propria) with inflammatory cells and cystic dilated glands are present, accompanied by cubic or columnar lining epithelium. They are distinguished from sporadic JPs (to which they are similar) by a smaller number of dilated glands and, simultaneously, a more significant number of smaller, proliferating glands. Another characteristic feature is the frequent neoplastic epithelial changes, which are much less common in sporadic polyps [157].

In 50–60% of JPs patients’ genomes, a germline mutation is found in *SMAD4* or *BMPR1A* tumor suppressor genes [158] involved in the BMP/TGF-β signaling pathway [159,160]. Most mutations are point mutations or small deletions of coding sequences, easily detected using sequencing screening methods. In contrast, about 15% of mutations are believed to be large deletions, affecting one or more exons and sometimes even the entire coding sequence, making identification difficult [158]. In the case of SMAD4 mutations, polyps are characterized by higher epithelial proliferation [161] and are more common in the upper GI tract. Additionally, mutation of this gene is more likely to cause gastric cancer [162]. In the remaining patients (50–40%), no *SMAD4* or *BMPR1A* gene mutations are detected, strongly suggesting the involvement of other potential mutations in the occurrence and pathogenesis of JPS.

The molecular basis of tumorigenesis in JPS is not yet well studied. One proposal suggests that BMPR1A is a so-called “landscaper”, which means that a damaged version of this gene creates a microenvironment that promotes the survival of cancer cells. This assumption is based on observing genetic alterations at the *BMPR1A* locus (10q22), predominantly found in the JP stroma. That implies that cancer arises from the pathological development of the stroma, leading to neoplastic transformation of the nearby epithelium [163]. Studies in mice have shown that inhibition of the expression of *BMP-4*, a participant in the BMP pathway, led to a JPS-like phenotype. The *BMP-4* is expressed exclusively in the mesenchymal compartment of the intestine, and its inhibition conditions the formation of polyps [164]. Another independent theory suspects SMAD4 of playing a role of a so-called “gatekeeper.” In other words, the expression of the protein product ensures control of cell growth and proliferation. When the expression is inhibited, excessive proliferation can lead to the development of cancer. Indeed, it has been discovered that homozygous deletions of *SMAD4* in JPS patients are limited only to JPs. The same situation occurs in a mouse model undergoing Smad4 knockout [165]. Both hypotheses are promising and not mutually exclusive.

Diagnosis and prophylaxis are essential parts of the fight against JPS. When the family history of the disease and the causing mutation are known, genetic testing of all members is recommended. In cases of the absence of the mutation, the person in question is not at risk. On the other hand, detecting the mutation calls for regular intestinal testing of such a person. When genetic testing is not available, it is recommended to have the first endoscopic examination at the age of 15 (or earlier if JPS symptoms appear). The procedure should be repeated every 2–3 years. Removal of JPs—a polypectomy—is an optional prophylactic procedure. Contraindications for colon polypectomy include a high number of polyps (>50–100), severe intestinal bleeding, diarrhea, polyps with dysplasia, as well as a strong family history of CRC [154,166,167]. When the large size of the polyps prevents endoscopy, surgical removal is recommended [167]. Ultimately, there are various surgical options, but all carry risks, and each case should be considered separately.

No drugs to effectively treat patients suffering from JPS are available. There have been experiments with sulindac (market name Clinoril), a non-steroidal anti-inflammatory drug used to inhibit COX-2. The COX-2 expression is higher in JPS polyps than in sporadic JPs due in part to the size of the polyps [168]. Hence, the idea of treating/mitigating JPS with COX-2 inhibition. To date, there have been no thorough studies, but two cases of reconstructive proctocolectomy patients suffering from JPS have been documented. Reconstructive proctocolectomy is a procedure during which the colon and rectum are entirely removed, and a so-called ileal pouch is created from a fragment of the small intestine. After a successful proctocolectomy, a series of polypectomies of the ileal pouch was performed. Throughout the treatment period, patients were administered sulindac and were not observed developing new ileal pouch polyps [166].

#### 3.3.3. Cowden Syndrome

CS, also known as Cowden-1 syndrome (CS-1), is an autosomal dominant inherited disease characterized by HPs in the GI tract, oral cavity, and skin [169]. Polyps in the GI tract form mainly in the large intestine and can range from 1 mm to several cm in diameter. In 25–30% of cases of the disease, there are no polyps in the gastrointestinal tract [170]. These polyps can also occur in the thyroid, breast, uterus, and brain. Patients affected by CS often have a larger head circumference (macrocephaly) and problems with the circulatory system. Children typically have learning disabilities and developmental delays and are sometimes found to have autism spectrum disorders [171]. Suffering from CS is associated with an increased risk of developing cancers of the breast (82.5%), thyroid (35.2%), uterine endometrium (28.2%), kidney (33.6%), colon (9.0%), skin (6.0%), and bladder (3.0%) [170,172]. Furthermore, CS has been estimated to occur once every 200,000 live births [173,174].

After examining families diagnosed with CS, about 85% of patients were found to have a germline mutation of the *PTEN* gene, and when a similar study was conducted on a group of patients whose CS diagnosis was unclear or were diagnosed with CS-like diseases, *PTEN* mutation was found in about 25% of them. The gene was mapped to chromosome 10q22-23 [175]. The *PTEN* (phosphatase and tensin homolog) is a phosphatase with a suppressor role; it negatively regulates the PI3K-Akt/PKB-mTOR signaling pathway, which controls the cell cycle and limits proliferation [176]. Therefore, partial or complete loss of function/lack of *PTEN* expression significantly increases the risk of tumorigenesis. The *PTEN* mutations are a major factor linking several disease entities, including Bannayan–Riley–Ruvalcaba syndrome (BRRS), which is considered allelic to CS, Proteus syndrome (PS), and CS [177,178]. These diseases are now classified as PTEN hamartoma tumor syndromes (PHTS). Furthermore, *PTEN* mutations (germline) have been reported in all 9 exons and are not limited to 1 or 2 types. Large deletions and duplications, small deletions, and insertions causing missense, nonsense, and frameshift mutations have been described. The most common nonsense mutations occur in exons 5, 7, or 8 [179]. A hot spot of Alu element insertions has been identified in exon 5 [180]. Pathogenic mutations of the *PTEN* promoter negatively affecting standard transcription and translation have also been defined [181]. Intron variants can cause abnormal alternative splicing and exon skipping [182]. Mutations of other genes can also cause CS, either indirectly modulating *PTEN* expression or affecting the PI3K-Akt/PKB-mTOR pathway in different ways. Mutations in the *SDHB* and *SDHD* genes have been reported to be associated with the so-called Cowden-2 syndrome (CS-2) and Cowden-3 syndrome (CS-3) [183].

Diagnosis and treatment of CS are based on the early discovery of the disease and regular examinations to detect malignant lesions as early as possible and remove them. The easiest symptom to spot and examine is macrocephaly in infants [171]. Genetic testing is essential; most panels allow *PTEN* to be tested for mutations. In addition, any family whose member is affected by CS should be tested and be under the care of specialists and genetic counselors. The most common risk of CS is the development of female breast cancer. It is recommended to have a breast examination every six months, starting at age 25. Once a woman reaches the age of 30–35, she should have her breasts examined by mammogram and MRI every year. There is also the option of prophylactic mastectomy, which reduces the risk of breast cancer by 90% [184].

Potential therapies for treating CS and preventing the disease’s development are underway. A mouse model of the disease has been established—PTEN deletion in the epithelium caused neoplastic lesions characterized by hyperproliferation, resembling those found in patients affected by CS. The researchers inhibited mTOR kinase using rapamycin, which led to the regression of the mucocutaneous lesions caused by the deletion. They also proved that using rapamycin before the disease progresses can stop the formation of lesions and, thus, prolong the life of the mice [185]. It is unknown whether rapamycin therapy can help CS patients; however, research like the one mentioned above offers hope.

## 4. Discussion

In recent years, there has been a significant increase in the incidence of colorectal cancer worldwide. Due to the non-specific symptoms and sometimes their complete absence in the early stages of cancer, diagnosis is critical. A genetic basis, usually inherited, is responsible for many CRC cases. Through tests that analyze specific mutations present in genes, it is possible to identify the disease affecting the patient (Table 1). On this basis, it is possible to distinguish several diseases characterized by the formation of polyps that can directly undergo malignant transformation. Clinical features, such as the average age of diagnosis, the number and type of polyps, and the possible location of tumors for each syndrome, are presented in Table 2. An important aspect is that in the course of those syndromes, not only colorectal cancer occurs, but also cancers located in entirely different areas. That is due to metastasis and the genetic nature of germline changes. The percentage risk of cancers associated with hereditary diseases is presented in Table 3. Due to the variety of clinical features of the conditions presented here and the type of symptoms, as well as their severity, the average age of diagnosis of cancers caused by individual disease entities varies, as shown in Table 4.

## 5. Conclusions

Hereditary predispositions to colorectal cancer are a group of heterogeneous diseases in which some symptoms overlap. However, differences in age and symptoms are observed in carriers of the same mutation and members of the same family. Therefore, their proper diagnosis is insufficient to rely on an analysis of pedigree and clinical data only. The use of molecular and immunohistochemical techniques is indispensable for comprehensively diagnosing the disease and implementing effective treatment.

## Figures and Tables

**Table 1:** Mutations in genes responsible for the described syndromes’ development [31,70,99,117,122,137,158,175].

Syndrome	Gene Mutations
LS	*MSH2*, *MLH1*, *MSH6*, *PMS2*
CMMRDS	*MLH1*, *MSH2*, *MSH6*, *PMS2*
FAP	*APC*
AFAP	*APC*
MAP	*MUTYH (MYH)*
NAP	*NTHL1*
PJS	*LKB1 (STK11)*
JPS	*SMAD4*, *BMPR1A*
CS	*PTEN*

Abbreviations are as follows: AFAP—attenuated familial adenomatous polyposis, CMMRDS—constitutional mismatch repair deficiency syndrome, CS—Cowden syndrome, FAP—familial adenomatous polyposis, JPS—juvenile polyposis syndrome, LS—Lynch syndrome, MAP—*MUTYH* (MYH)-associated polyposis, NAP—*NTHL1*-associated polyposis, PJS—Peutz–Jeghers syndrome.

**Table 2:** Clinical characteristics of syndromes [15,122,186,187,188,189,190,191,192].

Syndrome	Average Age of Diagnosis	Number of Polyps	Type of Polyps	Location of Tumors
LS	About 50 years	From 1 to several	Adenomatous	Endometrium, stomach, bile ducts, urinary tract, ovaries
CMMRDS	Before 18 years	From 1 to several	Adenomatous	Lymphatic system, brain and central nervous system, colon, rectum, duodenum, jejunum, ileum, uterus, bladder, ureter
FAP	15 years	Numerous (more than 100, mostly uncountable)	Adenomatous	duodenum, fundus of the stomach, liver, adrenal gland, soft tissues, brain, thyroid, bones
AFAP	20 years	Up to 100	Adenomatous	-
MAP	30–40 years	Up to 100	Adenomatous	-
NAP	55 years	Up to 100	Adenomatous	Breast, reproductive organs, bladder, skin
PJS	Several years	Several	Hamartomatous	Pancreas, breast, lungs, ovaries, testicles
JPS	10–30 years	Several	Hamartomatous	-
CP	Several years	Few	Hamartomatous	Thyroid, bladder, kidneys, breast, nipples, a body of the uterus

Abbreviations are as follows: AFAP—attenuated familial adenomatous polyposis, CMMRDS—constitutional mismatch repair deficiency syndrome, CS—Cowden syndrome, FAP—familial adenomatous polyposis, JPS—juvenile polyposis syndrome, LS—Lynch syndrome, MAP—*MUTYH* (MYH)-associated polyposis, NAP—*NTHL1*-associated polyposis, PJS—Peutz–Jeghers syndrome.

**Table 3:** Percentage risk of cancers associated with hereditary syndromes [15,134,154,170,172,192,193,194,195,196].

Organ/Percentage Risk of Cancers	LS	CMMRDS	FAP	AFAP	MAP	PJS	JPS	CS
Colon	50–70%	25%	Up to 100%	70%	43–63%	39%	38,7%	9%
Duodenum	—	8%	3–5%	4–12%	4%	—	—	—
Bladder	—	1%	—	—	6–25%	—	—	3%
Stomach	7%	—	5%	—	1%	29%	—	—
Ovary	9%	—	—	—	6–14%	21%	—	—
Liver		—	2%	—	—	—	—	—
Urinary tract	3%	—	<1–25%	—	—	—	—	—
Small intestine	3%	8%	3–10%	4–12%	—	13%	—	—
Brain	3%	53%	2%	—	—	—	—	—
Pancreas	4%	—	1.7%	—	—	36%	—	—
Prostate	—	—	—	—	—	—	—	—
Breast	—	—	—	—	—	54%	—	82.5%
Thyroid	—	—	2%	1–2%	—	—	—	35.2%
Uterus	40–60%	4%	—	—	—	9%	—	28.2%
Cervix	—	—	—	—	—	10%	—	—
Testis	—	—	—	—	—	9%	—	—
Lungs	—	—	—	—	—	15%	—	—
Skin	—	—	—	—	—	—	—	6%
Lymphatic system	—	31%	—	—	—	—	—	—

Abbreviations are as follows: AFAP—attenuated familial adenomatous polyposis, CMMRDS—constitutional mismatch repair deficiency syndrome, CS—Cowden syndrome, FAP—familial adenomatous polyposis, JPS—juvenile polyposis syndrome, LS—Lynch syndrome, MAP—*MUTYH* (MYH)-associated polyposis, NAP—*NTHL1*-associated polyposis, PJS—Peutz–Jeghers syndrome.

**Table 4:** The average age of diagnosis of cancers associated with hereditary syndromes [15,134,154,192,194,195,197].

Organ/Average Age of Diagnosis of Cancers	LS	CMMRDS	FAP	AFAP	MAP	PJS	JPS	CS
Colon	45	16	40	55	40–60	45.8	43.9	47
Duodenum	—	28	44	60	61	—	—	—
Bladder	—	20	—	—	61	—	—	—
Stomach	49–55	—	49	—	38	30.1	54	—
Ovary	42–54	—	—	—	51	28	—	—
Liver	54–57	—	<5	—	—	—	—	—
Urinary tract	52–57		—	—	—	—	—	40
Small intestine	46–51	28	44	60	—	41.7	—	—
Brain	50–55	9	15–21	—	—	—	—	—
Pancreas	51.5–56.5	—	50	—	—	40.8	—	—
Prostate	59–60	—	—	—	—	—	—	—
Breast	46–52	—	—	—	—	37	—	38–46
Thyroid	—	—	25–33	26	—	—	—	31–38
Uterus	—	28	—	—	—	43	—	—
Cervix	—	—	—	—	—	34.3	—	—
Testis	—	—	—	—	—	8.6	—	—
Lungs	—	—	—	—	—	47	—	—
Lymphatic system	—	6	—	—	—	—	—	—

Abbreviations are as follows: AFAP—attenuated familial adenomatous polyposis, CMMRDS—constitutional mismatch repair deficiency syndrome, CS—Cowden syndrome, FAP—familial adenomatous polyposis, JPS—juvenile polyposis syndrome, LS—Lynch syndrome, MAP—*MUTYH* (MYH)-associated polyposis, NAP—*NTHL1*-associated polyposis, PJS—Peutz–Jeghers syndrome.

## Data Availability

Data are available in publicly accessible databases. The data presented in this study are openly available in the Medline and PubMed databases and on the publisher’s website. The keywords that were used: colorectal, adenoma, cancer risk, familial adenomatous polyposis, *MUTYH*-associated polyposis, Muir–Torre syndrome, *NTHL1*-associated polyposis, Peutz–Jeghers syndrome, juvenile polyposis syndrome, Cowden syndrome, and attenuated familial adenomatous polyposis. All data in the text are quoted and all works used are listed in the bibliography along with the DOI and reference numbers.
